# Hypothermic Machine Perfusion Is Associated with Improved Short-Term Outcomes in Liver Transplantation: A Retrospective Cohort Study

**DOI:** 10.3390/life15071112

**Published:** 2025-07-16

**Authors:** Alexandru Grigorie Nastase, Alin Mihai Vasilescu, Ana Maria Trofin, Mihai Zabara, Ramona Cadar, Ciprian Vasiluta, Nutu Vlad, Bogdan Mihnea Ciuntu, Corina Lupascu Ursulescu, Cristina Muzica, Irina Girleanu, Iulian Buzincu, Florin Iftimie, Cristian Dumitru Lupascu

**Affiliations:** 1 Faculty of Medicine, Grigore T. Popa University of Medicine and Pharmacy, 700115 Iasi, Romania; alex.gr.nastase@gmail.com (A.G.N.); mihai-lucian.zabara@umfiasi.ro (M.Z.); tibaramona@yahoo.com (R.C.); nutu.vlad@gmail.com (N.V.); bogdan.m.ciuntu@yahoo.com (B.M.C.); corina.ursulescu@gmail.com (C.L.U.); cristina-maria.muzica@umfiasi.ro (C.M.); gilda_iri25@yahoo.com (I.G.); or rectorat@umfiasi.ro (I.B.); iftimienicolaeflorin@yahoo.com (F.I.); cristian.lupascu@yahoo.com (C.D.L.); 2 General Surgery and Liver Transplant Clinic, St. Spiridon University Hospital, 700111 Iasi, Romania; vascip25@yahoo.com; 3 Radiology Clinic, St. Spiridon University Hospital, 700111 Iasi, Romania; 4 Institute of Gastroenterology and Hepatology, Sf. Spiridon University Hospital, 700111 Iasi, Romania; 5 Anesthesiology and Intensive Care Department, Sf. Spiridon University Hospital, 700111 Iasi, Romania

**Keywords:** liver transplantation, hypothermic machine perfusion, ischemia-reperfusion injury

## Abstract

Introduction: Liver transplantation remains the definitive treatment for end-stage liver disease but faces critical challenges including organ shortages and preservation difficulties, particularly with extended criteria donor (ECD) grafts. Hypothermic machine perfusion (HMP) represents a promising alternative to traditional static cold storage (SCS). Methods: This retrospective study analyzed outcomes from 62 liver transplant recipients between 2016 and 2025, comparing 8 grafts preserved by HMP using the Liver Assist^®^ system and 54 grafts preserved by SCS. Parameters assessed included postoperative complications, hemodynamic stability, ischemia times, and survival outcomes. Results: HMP significantly reduced surgical (0% vs. 75.9%, *p* = 0.01) and biliary complications (0% vs. 34.4%, *p* = 0.004), improved hemodynamic stability post-reperfusion (∆MAP%: 1 vs. 21, *p* = 0.006), and achieved superior one-year survival rates (100% vs. 84.4%). Despite longer ischemia periods, grafts treated with HMP exhibited fewer adverse effects from ischemia-reperfusion injury. Discussion: These findings highlight the substantial benefits of HMP, particularly in improving graft quality from marginal donors and reducing postoperative morbidity. Further adoption of this technology could significantly impact liver transplantation outcomes by expanding the viable donor pool. Conclusions: The study underscores the effectiveness of hypothermic machine perfusion (HMP) as a superior preservation method compared to traditional static cold storage (SCS), HMP appears to be associated with improved short-term outcomes in liver transplantation. By substantially reducing postoperative complications and enhancing graft viability, HMP emerges as a pivotal strategy for maximizing the use of marginal donor organs. Further research and broader clinical implementation are recommended to validate these promising results and to fully harness the potential of HMP in liver transplantation.

## 1. Introduction

Liver transplantation is the definitive therapeutic approach for patients with end-stage liver disease, hepatocellular carcinoma, and selected other hepatic disorders [1]. Despite significant advances in surgical techniques and immunosuppressive regimens achieving approximately 90% one-year survival rates, a persistent shortage of suitable donor organs continues to present significant challenges [2]. To address this gap, the utilization of extended criteria donors (ECD) and donation after circulatory death (DCD) has become increasingly common. However, these types of grafts are particularly vulnerable to ischemia-reperfusion injury (IRI), early allograft dysfunction (EAD), and severe biliary complications [3,4].

The historical foundations of machine perfusion trace back to early 19th-century physiological studies, starting with Cesar Julien Jean Le Gallois who theorized maintaining organ viability via artificial circulation—laying the groundwork for extracorporeal circulation techniques [5]. Subsequent experiments by Loebell in 1849 demonstrated successful perfusion of isolated pig kidneys [6]. Significant advancements followed, notably the closed-loop circulation system developed by von Frey and Gruber in 1885, and Jacobj’s dual-circuit system in 1895 which employed an isolated lung for oxygenation, substantially extending perfusion times [5,7].

The modern era of machine perfusion began in the 1960s with Folkert Belzer’s introduction of hypothermic machine perfusion (HMP), significantly improving kidney graft preservation and enabling successful human transplantation by 1967 [8]. Although initially perceived as technically complex and costly, the development of the Collins solution in 1969, an intracellular preservation medium, standardized and popularized static cold storage (SCS), a simpler and more economical approach to organ preservation [9,10]. The widespread adoption of the University of Wisconsin (UW) solution during the 1980s further entrenched SCS as the primary preservation method [11,12]. However, limitations became increasingly apparent, particularly in marginal donor organs, prompting renewed interest in HMP, exemplified by a pivotal 2010 study demonstrating its safety and efficacy in liver graft preservation [13,14].

Although SCS remains extensively used due to simplicity and cost-effectiveness, it inherently suffers from limitations, including progressive ischemic injury and mitochondrial dysfunction, particularly problematic in marginal grafts [15,16]. Consequently, machine perfusion methods have garnered renewed interest as potential alternatives to mitigate ischemic damage and improve outcomes [17].

In accordance with the criteria established by Eurotransplant, extended criteria donors (ECD) were defined by the presence of one or more donor characteristics associated with a heightened risk of post-transplant complications, including primary nonfunction and early allograft dysfunction. These criteria specifically included donor age greater than 65 years, intensive care unit (ICU) stay longer than 7 days, body mass index (BMI) exceeding 30 kg/m^2^, hepatic steatosis exceeding 30%, serum sodium concentration greater than 165 mmol/L, alanine aminotransferase (ALT) levels three times normal value, aspartate aminotransferase (AST) levels levels three times normal value, and total serum bilirubin greater than 3 mg/dL. Hepatic steatosis was assessed intraoperatively by an experienced retrieval surgeon, and grafts were categorized as steatotic when macrovesicular steatosis was estimated to exceed 30%. It is important to note that grafts meeting these extended criteria are particularly susceptible to ischemia-reperfusion injury, a limitation inadequately addressed by static cold storage (SCS), thus highlighting the need for alternative preservation methods such as machine perfusion [18].

Variants of HMP, specifically Hypothermic Oxygenated Perfusion (HOPE) and Dual Hypothermic Oxygenated Perfusion (DHOPE), are promising techniques integrating active perfusion with oxygen delivery at low temperatures (≤12 °C) [19]. HOPE utilizes oxygenated perfusion solely through the portal vein, whereas DHOPE involves simultaneous perfusion through both the portal vein and hepatic artery, ensuring enhanced oxygenation of critical structures, notably the bile duct [20]. These techniques significantly reduce mitochondrial damage, limit ischemic cell injury, facilitate metabolic waste clearance, and lower reactive oxygen species production upon reperfusion [21]. HOPE, in particular, has shown marked efficacy in reducing ischemia-related complications in marginal and DCD grafts, suggesting potential for broader clinical use and expansion of the donor pool [22]. Additional research highlights HMP’s immunomodulatory properties, potential to mitigate acute graft rejection, and possible impact on reducing cancer recurrence, underscoring its comprehensive benefits in transplantation contexts [23,24].

Despite these advantages, global clinical adoption of HMP remains uneven due to differences in resource allocation, logistical challenges, and clinical practice guidelines [25]. Nevertheless, accelerated adoption in the United States, driven by accumulating evidence supporting improved outcomes and increased organ utilization, emphasizes the growing clinical relevance of machine perfusion strategies [26,27].

## 2. Materials and Methods

### 2.1. Study Design and Patient Selection

This retrospective study analyzed the patients who underwent liver transplantation from brain-dead donors (DBD) at the Liver Transplant Center in Iași, Romania, from 2016 to 2025. Eligible patients were adults (>18 years old) who received liver grafts from DBD donors. Recipients of living donor liver transplants and those under 18 years old were excluded.

### 2.2. Study Groups

**Figure 1:**
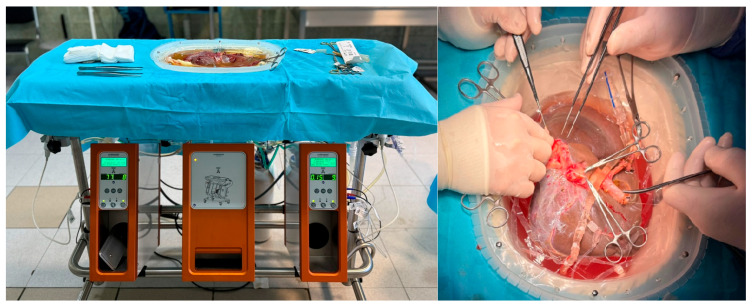
Hipothermic machine perfusion of the liver graft—Liver Assist^®^ system (Organ Assist, Groningen, The Netherlands).

Patients were categorized into two groups based on liver graft preservation techniques:

- Static Cold Storage (SCS): conventional method of preservation.
- Hypothermic Machine Perfusion (HMP): was performed utilizing the Liver Assist^®^ system (Organ Assist, Groningen, The Netherlands) (Figure 1), applied exclusively to grafts from donors meeting extended criteria (ECD). Specific indications in this cohort included donor age > 65 years, moderate-to-severe liver steatosis assessed intraoperatively by frozen biopsy, elevated serum transaminase levels (>3 times the normal upper limit), prolonged intensive care unit (ICU) stay (>7 days), prolonged cold ischemia time (>6 h) or anticipated ischemia time > 6 h due to logistical delays.
- The perfusion protocol was initiated immediately upon graft arrival at the transplant center, maintaining perfusion temperatures at 8–12 °C. Flow rates were set at 150–300 mL/min for the portal vein and 50–150 mL/min for the hepatic artery, with perfusion pressures held at 3–5 mmHg (portal vein) and 25–30 mmHg (hepatic artery). Oxygenation was delivered using a gas mixture of 100% O_2_. Perfusion duration was standardized to 2 h for 6 patients and extended to 3 h for 2 patients.

### 2.3. Data Collection and Variables

Collected variables included demographic and clinical characteristics of donors and recipients, intraoperative and postoperative data, and biochemical markers measured on postoperative day 7 (bilirubin, INR, AST, ALT, potassium, bicarbonate, calcium). Additional parameters analyzed included MELD scores, ischemia times (warm, cold, and total ischemia), early postoperative complications, normalized difference of mean arterial pressure (measured 5 min before and 5 min after venous reperfusion) (∆MAP%), surgical complications, and patient survival.

Post-reperfusion syndrome was also assessed, defined by as a decrease in mean arterial pressure (MAP) greater than 30% below the baseline value, lasting for at least 1 min, occurring during the first 5 min after reperfusion of the liver graft [28].

As previously described, early allograft dysfunction (EAD) was defined by the presence of one or more of the following variables: total bilirubin ≥ 10 mg/dL (171 μmol/L) on postoperative day 7, INR ≥ 1.6 on postoperative day 7, or ALT or AST > 2000 IU/mL within the first 7 postoperative days [29].

Postoperative outcomes were assessed with a focus on the incidence and type of complications. Specifically, we evaluated surgical complications, including biliary and vascular complications (venous thrombosis), as well as postoperative hemorrhage. Each complication was recorded and analyzed based on its occurrence, expressed as absolute numbers (n) and percentages (%).

### 2.4. Statistical Analysis

Data were analyzed using IBM SPSS Statistics (version 26). Continuous variables were tested for normal distribution and presented as means ± standard deviations (SD). Comparisons between SCS and HMP groups were conducted using independent samples *t*-tests or Mann–Whitney U tests depending on data normality. Categorical variables were analyzed using the chi-square test or Fisher’s exact test. Statistical significance was defined at a *p*-value < 0.05. Missing data were managed by the listwise deletion method.

The study protocol was reviewed and approved by the Institutional Review Board of St. Spiridon University Hospital in accordance with national ethical guidelines. Due to the retrospective nature and use of anonymized data, the requirement for informed consent was waived.

## 3. Results

### 3.1. Preoperative Parameters

A total of 62 liver transplant recipients were included: 54 in the SCS group and 8 in the hypothermic machine perfusion (HMP) group. The mean recipient age was slightly lower in the HMP group (42.75 ± 8.53 years) compared to SCS (47.90 ± 9.2 years), without statistical significance (*p* = 0.14). A male predominance was observed in both groups (60.3% in SCS vs. 75% in HMP, *p* = 0.18). MELD scores at the time of transplantation were also similar, though lower in the HMP group (15.33 ± 1.86) versus SCS (18.43 ± 5.78, *p* = 0.19).

The distribution of etiologies was comparable between groups (*p* = 0.85). HBV + HDV co-infection was the most common cause in both groups (36.2% SCS vs. 50% HMP). Other causes included toxic cirrhosis, autoimmune hepatitis, Wilson disease and malignancy-related liver disease (hepatocellular carcinoma (HCC) and cholangiocarcinoma) (Table 1).

All eight grafts preserved using Hypothermic Machine Perfusion (HMP) were retrieved from extended criteria donors (ECD), based on predefined clinical and histological parameters. The most frequently encountered ECD criteria were donor age > 65 years (4 out of 8 cases) and liver steatosis > 30% confirmed via intraoperative frozen section (3 cases). Elevated serum transaminase levels (one case), prolonged intensive care unit (ICU) stay (one case), and prolonged cold ischemia time were each present in two donors (Table 2). Notably, several grafts exhibited more than one risk factor, supporting the selective application of HMP in high-risk scenarios. This stratification further emphasizes the rationale for perfusion-based preservation when conventional static cold storage may be insufficient to mitigate the ischemia-reperfusion injury risk inherent to marginal grafts.

### 3.2. Intraoperative and Ischemia Parameters

The mean operative time was comparable between the groups (SCS: 494.91 ± 75.26 min vs. HMP: 487.50 ± 79.78 min, *p* = 0.79). Estimated blood loss was lower in the HMP group (5200 ± 1307 mL) compared to SCS (6881.82 ± 4779.78 mL), although this difference was not statistically significant (*p* = 0.30).

Total ischemia time was significantly longer in the HMP group (368.00 ± 57.75 min vs. 287.96 ± 73.14 min, *p* = 0.004), driven primarily by prolonged cold ischemia (310.25 ± 56.87 min vs. 231.33 ± 70.65 min, *p* = 0.004). Warm ischemia time was equivalent between groups (*p* = 0.79).

### 3.3. Hemodynamic Response and Post-Reperfusion Markers

The drop in mean arterial pressure immediately after reperfusion was significantly lower in the HMP group (1 ± 1.5) compared to SCS (21 ± 9.6, *p* = 0.006), suggesting improved hemodynamic stability.

No patients in either group exceeded thresholds for EAD-defining values of bilirubin or transaminases. However, two patients in the SCS group exhibited ALT > 2000 U/L by day 7. No patient in the HMP group crossed this threshold (Table 3).

### 3.4. Postoperative Parameters

- Early Graft Function and Biochemical Markers (Day 7)

Biochemical values measured on postoperative day 7 were generally comparable. AST levels were significantly higher in the SCS group (105.25 ± 13.45 U/L) than in the HMP group (74.23 ± 70.97 U/L, *p* = 0.04). Total bilirubin was lower in HMP (1.50 ± 0.95 mg/dL vs. 2.59 ± 1.59 mg/dL, *p* = 0.06), though not statistically significant. Other parameters, including INR, potassium, calcium, bicarbonate, and ALT, showed no significant differences (Table 4).

- Complications

Surgical complications were significantly more frequent in the SCS group (75.9%) compared to none in the HMP group (*p* = 0.01). Biliary complications were particularly pronounced: 34.4% in SCS vs. 0% in HMP (*p* = 0.004). Rates of vascular complications (portal vein throbosis (n = 10) and inferior vena cava thrombosis (n = 1) (13.7% vs. 0%, *p* = 0.19) and hemorrhagic events (18.9% vs. 0%, *p* = 0.25) did not reach statistical significance.

Patients who received liver grafts preserved by Hypothermic Machine Perfusion (HMP) had a shorter mean hospital stay (21.0 ± 3.46 days) compared to those who received grafts preserved by Static Cold Storage (SCS) (27.71 ± 13.19 days). However, this difference did not reach statistical significance (*p* = 0.16). The lower standard deviation in the HMP group also indicates more consistent hospital stays among these patients compared to the SCS group.

### 3.5. Survival Outcomes

At one year post-transplant, 100% of patients in the HMP group remained alive compared to 84.4% in the SCS group. Although this difference did not reach statistical significance (*p* = 0.22), it is clinically relevant, especially considering that HMP was used exclusively for marginal grafts. The absence of early mortality and complications in the HMP group suggests that machine perfusion may help neutralize donor-related risk, contributing to excellent short-term survival.

In the SCS group, 9 out of 58 patients (15.6%) died within the first year, with most deaths occurring in patients who had experienced significant postoperative complications or early graft dysfunction.

**Figure 2:**
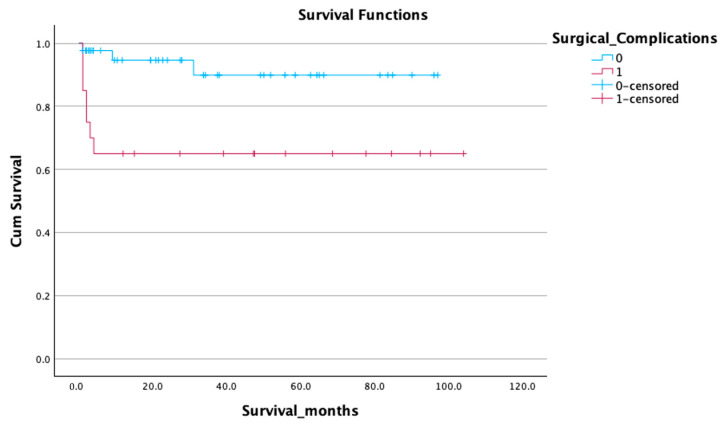
Impact of surgical complications survival curve—Kaplan-Meier.

Long-term survival analysis via Kaplan–Meier estimates showed:

A 5-year cumulative survival probability of 86.58% in the overall cohort. Stratification revealed that patients without surgical complications had significantly better 5-year survival (mean 89.08 ± 4.48 days) than those with complications (mean 68.38 ± 10.89 days, *p* = 0.003) (Figure 2).

**Figure 3:**
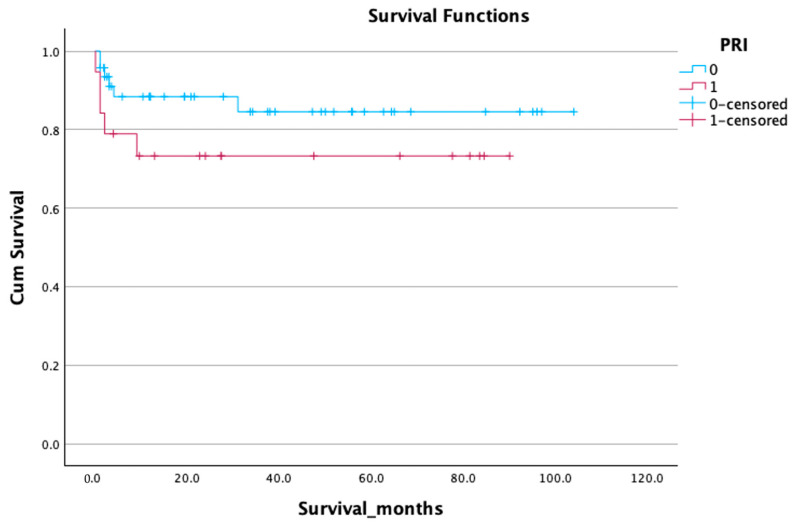
Impact of postreperfusion injury survival curve—Kaplan-Meier.

Although the presence of a positive Post-Reperfusion Injury Index (PRI) was associated with a trend toward lower survival (66.79 ± 8.96 days vs. 89.50 ± 5.56 days), this difference did not reach statistical significance (*p* = 0.129) (Figure 3) (Table 5).

These findings suggest that:

HMP offers a survival advantage at 1 year, especially in the context of extended criteria donors.

Complication-free perioperative course, more frequently observed in the HMP group, correlates with better long-term survival.

Longer-term follow-up beyond the first year was not uniformly available across all patients. Future updates will include 3- and 5-year outcome reporting.

5-year outcomes are influenced more by early surgical events and graft injury than by preservation strategy alone, highlighting the importance of both graft conditioning and meticulous postoperative management.

## 4. Discussion

In liver transplantation, the choice of preservation and perfusion techniques significantly influences graft viability and patient outcomes.

### 4.1. HMP vs. SCS

The utilization of ECD organs is a cornerstone strategy to expand the donor liver pool and address the persistent organ shortage [18]. However, these grafts are inherently more susceptible to PRI, leading to higher rates of EAD, postoperative complications, particularly biliary issues, and potentially inferior long-term outcomes [30]. HMP technologies, such as the Liver Assist^®^ system employed in this study, aim to mitigate these risks by providing continuous, oxygenated perfusion at low temperatures. This approach reduces the grafts metabolic demands, facilitates the clearance of accumulated metabolic waste products, supplies oxygen to maintain cellular energy stores and may even allow for a degree of cellular repair prior to implantation [31].

The Liver Assist^®^ device used in this study is capable of performing DHOPE, a technique involving perfusion of both the portal vein and the hepatic artery [32]. DHOPE is specifically recognized for its potential to enhance oxygenation of the bile ducts. Given that the HMP group consisted entirely of ECD grafts, which often carry increased risks of biliary problems, the observed 0% incidence of biliary complications points towards HMP, potentially through DHOPE, effectively mitigating IRI in these delicate structures. This resulted in a notable reduction in post-transplant morbidity [33].

Several meta-analyses consistently report on key outcomes such as EAD, biliary complications, including NAS, and graft survival rates. Regarding EAD, HMP significantly reduces the rate of EAD compared to SCS, with an odd ratio (OR) of 0.51 (95% CI 0.34–0.76; *p* = 0.001; *I*^2^ = 0%) [34]. This finding is echoed in another meta-analysis which demonstrated that SCS is associated with a higher incidence of EAD with a *p*-value of <0.00001 [35]. Similarly, a significantly reduced incidence of EAD with machine perfusion in general compared to SCS (OR = 0.46; 95% CI = 0.31–0.67; *p* < 0.0001) was present in a different meta-analysis, with subgroup analysis indicating this benefit extends to HMP [36]. Furthermore, a study mentioned that EAD incidence rates were 27% in the HMP group (risk ratio [RR], 0.43; 95% CI, 0.20–0.91) versus 40% in the SCS control group (RR, 0.61; 95% CI, 0.39–0.96) [28]. These consistent findings across multiple studies strongly suggest that HMP offers a protective effect against the development of early allograft dysfunction following liver transplantation.

The HMP group in this study received grafts meeting specific high-risk ECD criteria, including factors like advanced donor age, steatosis, and prolonged ischemia times. The exceptional outcomes in this cohort, appearing superior to the SCS group which likely included grafts of varying, and potentially lower average, risk, suggest that HMP may offer more than just marginal improvements. It may fundamentally alter the post-transplant trajectory of organs previously considered too high-risk for routine use. Broader application of such effective HMP strategies could therefore lead to a significant expansion of the usable donor pool, increase organ utilization rates and ultimately contribute to reducing waitlist mortality.

While not all studies explicitly report NAS, HMP reduces the rate of non-anastomotic biliary strictures compared to SCS (OR 0.34; 95% CI; 0.17–0.67; *p* = 0.002, *I*^2^ = 0%) [34]. A significantly lower incidence of total biliary complications and ischemic cholangiopathy with mechanical perfusion particularly HMP, which are related to the concept of NAS was reported (OR 0.39; 95% CI; 0.18–0.85; *p* = 0.02) [36]. These results indicate a potential advantage of HMP in mitigating biliary complications that can significantly impact graft function and patient outcomes.

The striking reduction in overall surgical complications (0% in HMP vs. 75.9% in SCS, *p* = 0.01) and the complete absence of biliary complications (0% in HMP vs. 34.4% in SCS, *p* = 0.004) in the HMP group are standout findings of this study [37]. This aligns with a growing body of evidence supporting the protective effects of HMP, particularly oxygenated variants like HOPE (Hypothermic Oxygenated Perfusion) and DHOPE, against IRI and its downstream consequences [27,32,38]. DHOPE, which ensures oxygen delivery via both the portal vein and hepatic artery, is specifically highlighted for its superior perfusion and oxygenation of the biliary tree, a structure highly susceptible to ischemic damage and a common source of morbidity after transplantation of marginal grafts [39]. The Liver Assist^®^ device used in this study is capable of DHOPE, making it plausible that this dual perfusion contributed to the observed excellent biliary outcomes. Numerous studies and meta-analyses have reported that HMP/HOPE can reduce EAD and biliary complications, including ischemic cholangiopathy, especially in DCD and ECD livers [40,41,42].

The HMP group demonstrated significantly less hemodynamic perturbation immediately upon graft reperfusion, with a mean arterial pressure drop (∆MAP%) of only 1.42% compared to 21.4% in the SCS group (*p* = 0.006). This suggests a substantial attenuation of Post-Reperfusion Syndrome (PRS). While no patients in either group met the formal EAD criteria based on day bilirubin or transaminase thresholds, it is noteworthy that two patients in the SCS group exhibited ALT levels exceeding 2000 U/L by day 7, a critical level not reached by any HMP patient. Additionally, total bilirubin levels on postoperative day 7 trended lower in the HMP group (1.50 ± 0.95 mg/dL vs. 2.59 ± 1.59 mg/dL, *p* = 0.06), further hinting at better early graft function.

PRS is a well-recognized clinical entity characterized by hemodynamic instability upon graft reperfusion, attributed to the release of potassium, inflammatory cytokines (e.g., TNF-α, IL-1β, IL-2, IL-8), and other vasoactive substances from the preserved graft into the recipients circulation. The severity of PRS is often exacerbated by the extent of IRI the graft has sustained [43].

A significant observation from this study is that the HMP group successfully utilized grafts despite experiencing significantly longer mean cold ischemia times (CIT): 310.25 ± 56.87 min for HMP vs. 231.33 ± 70.65 min for SCS, *p* = 0.004) and consequently longer total ischemia times (368.00 ± 57.75 min for HMP vs. 287.96 ± 73.14 min for SCS, *p* = 0.004). Prolonged CIT is a well-established and potent risk factor for increased IRI, EAD, biliary complications, and poorer graft survival when livers are preserved using SCS [44]. A primary rationale for developing machine perfusion technologies is precisely to mitigate the detrimental effects of ischemia, thereby making longer preservation times safer and potentially expanding organ sharing capabilities. Indeed, studies have demonstrated that HMP can safely extend preservation times without compromising outcomes [45].

At one year post-transplant, patient survival was 100% in the HMP group compared to 84.4% in the SCS group (*p* = 0.22). While this difference did not achieve statistical significance, likely due to the small sample size of the HMP cohort, a 15.6% absolute difference in survival is clinically compelling, particularly given that HMP was exclusively used for ECD grafts, which are typically associated with higher risks. Furthermore, the studys long-term survival analysis revealed that a complication-free perioperative course, which was significantly more frequent in the HMP group (100% vs. 24.1%), correlated significantly with better 5-year survival (mean 89.08 months for no complications vs. 68.38 months with complications, *p* = 0.003).

The trend towards improved survival with HMP is consistent with findings from other studies, which have associated HMP with improved graft survival, especially when used for ECD-DBD and DCD livers. The strong link observed in this study between minimizing early postoperative complications and enhancing long-term survival is a well-established principle in transplantation surgery. Early adverse events can set a negative trajectory for long-term graft function and patient health.

The combination of 100% one-year survival for HMP-treated ECDs and the 0% surgical and biliary complication rate in this high-risk group strongly suggests that HMP can effectively level the playing field for these marginal organs. ECDs are inherently at higher risk for poor outcomes with SCS. The HMP ECD group in this study not only avoided early mortality but also had a complication-free course, which the study itself links to superior 5-year survival. This implies that by preventing early complications in ECDs, HMP is directly paving the way for potentially superior long-term survival for these initially disadvantaged grafts, possibly yielding outcomes that may even surpass those of standard grafts preserved by SCS if the latter experience significant complications.

If HMP consistently translates to better long-term survival for ECDs by minimizing these critical early insults, it could shift the focus of transplant programs from mere organ utilization to optimizing the quality and longevity of transplanted marginal grafts. This would lead to more life-years gained per transplant, reduce the substantial physical, emotional, and economic burden of re-transplantation, and enhance the overall efficiency and impact of the transplant system by addressing not just organ quantity but also the quality of life and long-term healthcare resource consumption.

Multiple studies suggest that HMP, especially in the forms of HOPE and DHOPE, is linked to better 1-year graft survival outcomes than SCS, notably in livers from DCD and ECD -a meta-analysis showed that the incidence of post-transplantation 1-year survival was increased in HMP compared to SCS (OR 2.19; 95% CI; 1.14–4.20; *p* = 0.02) [46]. These findings were suported by another meta-analysis that reported an improved 1-year graft survival in HMP compared to SCS (OR 2.36; 95% CI; 1.55–3.62) [27]. Furthermore, a 2025 meta-analysis of randomized controlled trials also reported the improved 1-year graft survival rate (RR: 1.07; 95% CI; 1.01–1.14; *p* = 0.02) and decreased graft failure (RR: 0.38; 95% CI; 0.16–0.90; *p* = 0.03) compared to SCS [47]. According to a 2024 a long-term follow-up analysis of a multicenter randomized controlled trial, HOPE reduces late-onset morbidity and improves long-term graft survival [48].

Regarding the long-term outcomes of HOPE-treated DCD livers compared to un-treated DCD livers and DBD, the 5-year graft and patient survival rate (censored for tumor-related graft loss) was comparable between HOPE treated DCD livers and standard DBD livers, with a cumulative survival probability of 94% (*p* = 0.024), while untreated DCD liver transplants had a 78% survival probability (*p* = 0.222) [49]. A recent multicentre observational cohort study collected data from 1202 liver transplantations (66% DBD) preformed at 22 European centers, reporting 1, 3 and 5 year graft survival rates for DBD and DCD livers of 95%, 92% and 91% for DBD livers vs. 92%, 87% and 81% for DCD livers (*p* = 0.003), the graft loss due to primary non-function and ischemic cholangiopathy was 2.3% and 0.4% for DBD, and 5% and 4.1% for DCD, HOPE reaching the IDEAL-D stage 4, providing additional evidence for its adoption in routine clinical practice [50].

From a cost-effectiveness perspective, the shorter and more consistent length of hospital stay observed in the Hypothermic Machine Perfusion (HMP) group (21.0 ± 3.46 days) compared to the Static Cold Storage (SCS) group (27.71 ± 13.19 days) suggests potential economic advantages. Although the difference did not reach statistical significance (*p* = 0.16), the reduced variability and shorter duration of hospitalization associated with HMP may translate into lower healthcare resource utilization and decreased associated costs, particularly if replicated in larger studies. Future research with larger sample sizes and detailed economic analyses are needed to accurately quantify the potential financial benefits and assess the overall cost-effectiveness of implementing HMP in clinical practice.

An additional key consideration is the cost-efficiency of HMP in comparison to SCS. A 2024 study explored this through an economic assessment of DHOPE using the Liver Assist device in the context of DCD liver transplantation based on a multicenter randomized controlled trial. The analysis accounted for expenses tied to the transplant itself, hospitalization, readmissions, and outpatient care during the first year following transplantation. To estimate HMP-related costs, three scenarios were modeled: (1) machine perfusion costs alone, (2) previous costs plus personnel expenses, and (3) all prior costs combined with depreciation for a dedicated organ perfusion facility.

The findings showed that DHOPE was already cost-effective after just one procedure under the first scenario. In the second and third scenarios, cost-effectiveness was achieved with an annual case volume of 25–30 procedures. The study concluded that, relative to SCS, DHOPE offers a cost-effective alternative for DCD liver transplants, with notable reductions in healthcare expenditures during the first year post-transplant [51].

While multiple randomized controlled trials have established the benefits of HMP (HOPE and DHOPE) in reducing ischemia-reperfusion injury and improving transplant outcomes, most were conducted under tightly controlled conditions in high-volume centers. Our study provides complementary real-world evidence from a medium-volume Eastern European transplant center, highlighting the feasibility and impact of implementing HMP in a non-trial, resource-variable setting. This experience contributes to the growing global body of data supporting machine perfusion and may help inform adoption strategies in similar health system contexts.

### 4.2. HOPE vs. DHOPE

HOPE and DHOPE are hypothermic oxygenated perfusion techniques used in liver transplantation to improve graft preservation compared to static cold storage, with HOPE involving portal vein perfusion alone and DHOPE combining perfusion through both the portal vein and hepatic artery [44]. While DHOPE is designed to more closely replicate physiological hepatic perfusion—potentially offering enhanced protection to the bile ducts due to their predominant arterial blood supply—evidence from multiple studies indicates that the differences in clinical efficacy between DHOPE and HOPE are not uniformly significant [52].

A retrospective study published in 2025 involving 183 liver transplant recipients—90 treated with HOPE and 93 with DHOPE—reported no statistically significant differences between the groups in terms of 1-year patient survival (81.7% in the HOPE group vs. 81.7% in the DHOPE group, *p* = 0.990),similarly comparable 1-year death-censored graft survival (91.2%—HOPE vs. 93.3%—DHOPE, *p* = 0.893), incidence of NAS in 10.96% in the HOPE group vs. 8.22% in the DHOPE group (*p* = 0.574) or the hospital length of stay (the median was 29 days for both HOPE and DHOPE), indicating that incorporating hepatic artery perfusion in DHOPE does not consistently yield improvements in post-transplant outcomes across all measured parameters, although larger cohorts and long-term follow-up data may be needed to confirm this, in order to conclude that the additional cannulation of the hepatic artery may not be beneficial in the context of hypothermic machine perfusion [53].

However, one study indicated that DHOPE might offer specific advantages. A retrospective cohort study published in 2024 that analyzed the outcomes after orthotopic liver transplantation in 247 patients, with the donor organs procured using SCS, or subjected to end-ischemic HOPE or DHOPE, found that, while HOPE generally led to a reduction in biliary complications and associated surgical revisions compared to SCS, this effect was primarily associated with the use of DHOPE (Table 6). In this study, only DHOPE was significantly associated with a reduced need for surgical revision for biliary complications upon both uni and multivariable logistic regression (OR 0.336, *p* = 0.011), suggesting that, while both techniques are feasible for preconditioning donor liver grafts, DHOPE might be more effective in mitigating biliary complications requiring surgical revisions [54].

A call for more risk-matched outcome analyses between single and dual HOPE has also been made in April 2025 to better understand the nuances of each technique in different clinical scenarios [21].

#### 4.2.1. Strengths of the Study

This study possesses several strengths that enhance the relevance of its findings. Firstly, its focus on ECD liver grafts addresses a critical and pressing challenge in modern liver transplantation, where the optimization of marginal organ utilization is paramount for expanding the donor pool and reducing waiting list mortality. This targeted approach is arguably more impactful than demonstrating marginal benefits in standard criteria donors. Secondly, the utilization of a commercially available and recognized HMP device, the Liver Assist^®^ system (Organ Assist, Groningen, The Netherlands), enhances the potential reproducibility and generalizability of the findings. Standardization is crucial for comparing results across different studies and for establishing robust clinical guidelines. The use of such a standardized system could therefore accelerate regulatory approval and wider clinical adoption of HMP, fostering multicenter collaborations and evidence generation more readily than studies relying on highly customized or experimental perfusion setups. Thirdly, the study involved a comprehensive assessment of outcomes. Data collection encompassed a diverse range of parameters, including intraoperative hemodynamic stability (∆MAP%), detailed postoperative complication rates (categorized as surgical, biliary, vascular, and hemorrhagic), an array of biochemical markers at a defined postoperative timepoint, and both short-term (1 year) and indicative long-term (5-year via Kaplan-Meier based on complication status) survival data. This multifaceted approach provides a more holistic view of HMPs impact compared to studies focusing on a narrower set of endpoints.

#### 4.2.2. Limitations of the Study

Despite its valuable contributions, this study is subject to several limitations. The retrospective design is an inherent limitation, introducing the potential for unmeasured confounding variables and biases in patient selection, treatment allocation, and data collection that cannot be fully controlled for. Prospective, randomized designs are superior in minimizing such biases.

The small sample size in the HMP group (n = 8) compared to the SCS group (n = 54) significantly limits the statistical power to detect true differences for some outcomes, particularly those with lower event rates, such as specific types of complications or mortality.

This may explain why the notable 15.6% absolute difference in 1-year survival between the HMP and SCS groups did not reach statistical significance. Findings in such small cohorts can also be more susceptible to random variation.

Being a single-center experience, the findings may not be universally generalizable to other transplant centers with different patient populations, surgical expertise, perioperative management protocols, or logistical setups.

The study protocol dictated that HMP was specifically used for ECDs meeting certain predefined criteria, while the SCS group presumably included a wider range of graft quality, potentially including standard criteria donors or less risky ECDs. This non-random allocation makes direct comparison between the groups challenging, as the HMP group inherently started with organs of higher baseline risk. If the HMP organs were indeed of poorer average baseline quality than those in the SCS group, then the observed superior outcomes in the HMP group would be even more remarkable, strongly underscoring HMPs reconditioning capabilities.

The non-randomized nature of this study and the lack of donor-matching between groups present a risk of selection bias. The HMP group consisted solely of ECD grafts, whereas the SCS group included standard criteria donors, limiting the comparability of outcomes. Future studies should consider propensity score matching or multivariate adjustment to better control for confounders and isolate the independent effect of HMP on transplant outcomes.

## 5. Conclusions and Future Research Directions

The study underscores the effectiveness of HMP as a superior preservation method compared to traditional SCS, HMP appears to be associated with improved short-term outcomes in liver transplantation. By reducing postoperative complications and enhancing graft viability, HMP shows promise as a strategy for maximizing the use of marginal donor organs. Further research and broader clinical implementation are recommended to validate these promising results and to fully harness the potential of HMP in liver transplantation.

The promising findings of this study, alongside its limitations, highlight several important avenues for future research. Foremost is the need for prospective, multicenter randomized controlled trials (RCTs). Such trials, with adequate statistical power and standardized protocols, are essential to definitively establish the efficacy of HMP in improving short- and long-term graft and patient survival, reducing specific complications (particularly biliary strictures and EAD), and confirming these benefits across diverse ECD populations and DCD grafts [4,55].

A critical area for investigation stems from the paradoxical AST elevation observed in the HMP group. Dedicated studies are required to map the complete release kinetics of AST, ALT, and other liver enzymes (e.g., lactate dehydrogenase (LDH), gamma-glutamyl transferase (GGT)) into both the perfusate during HMP and the recipients serum at multiple, frequent timepoints post-transplantation (e.g., daily for the first week). HMP offers a unique window to not only treat but also assess the organ. Biomarkers measurable during or immediately after HMP could provide dynamic, organ-specific functional data, moving beyond static donor characteristics to facilitate more objective assessment of ECD graft quality after reconditioning, potentially leading to even safer expansion of the donor pool and more personalized transplant decisions [56].

Further research should also focus on optimizing HMP protocols. This includes comparative studies of different HMP parameters such as perfusion routes (HOPE vs. DHOPE), perfusion pressures and flow rates, oxygen concentrations, perfusate compositions (e.g., addition of cytoprotective agents), and the optimal duration of perfusion for various types of ECD grafts (e.g., steatotic livers, DCD organs, grafts from elderly donors) to tailor the technology for maximal benefit and cost-effectiveness [30,57].

Rigorous health economic analyses are also required. These studies should evaluate the overall economic impact of implementing HMP, considering the costs of devices and consumables against potential savings from reduced complication rates, shorter ICU and hospital stays, decreased need for readmissions and re-interventions, and potentially fewer re-transplantations.

## Figures and Tables

**Table 1:** Patient demographics.

Variable	SCS	HMP	*p*
Age Years, mean (SD)	47.90 (9.2)	42.75 (8.53)	0.14
Male Genger, n, (%)	35 (60.3)	6 (75)	0.18
Urban living environment, n, (%)	33 (56.9)	4 (50)	0.234
MELD Score, mean (SD)	18.43 (5.78)	15.33 (1.86)	0.19
PRI, n, (%)	19 (32.8)	0 (0)	0.451
Survival 1 year, n, (%)	49 (84.4)	8 (100)	0.22
Aetiology, n, (%) HVB HVB-D HVC Toxic Autoimmune Hepatitis Wilson disease NASH Budd Chiari Disease HCC Colangiocarcinoma	12 (20.7) 21 (36.2) 3 (5.2) 13 (22.4) 6 (10.3) 1 (1.7) 1 (1.7) 1 (1.7) 7 (12.1) 2 (3.4)	2 (25) 4 (50) 0 (0) 2 (25) 0 (0) 0 (0) 0 (0) 2 (25) 0 (0) 0 (0)	0.85
Complications, n, (%) Surgical complications Biliary complications Vascular complications Haemorrhage	44 (75.9) 20 (34.4) 8 (13.7) 11 (18.9) 13 (22.4)	2 (25) 0 (0) 0 (0) 0 (0) 0 (0)	0.01 0.004 0.19 0.25 0.12

**Table 2:** ECD donors characteristics.

Extended Criteria Donors (ECD)	P1	P2	P3	P4	P5	P6	P7	P8
Age > 65 years	x			x		x	x	
Liver steatosis > 30%			x	x				x
Elevated serum transaminase		x						
Prolonged ICU stay		x						
Prolonged cold ischemia time					x		x	

**Table 3:** Neohepatic parameters.

Variable	SCS	HMP	*p*
∆MAP%, n (SD)	21 (9.6)	1 (1.5)	0.006
INR—Day 7 > 1.6, n (%)	8 (13.6)	0 (0)	0.32
Total Bilirubin—Day 7 > 10 mg/dL, n (%)	0 (0)	0 (0)	0.06
AST—Day 7 > 2000 U/L, n (%)	0 (0)	0 (0)	0.15
ALT—Day 7 > 2000 U/L, n (%)	2 (3.4)	0 (0)	0.23

**Table 4:** Perioperative and postoperative parameters.

Variable	SCS Mean	SCS SD	HMP Mean	HMP SD	*p*-Value
Length of Hospital Stay (days)	27.71	13.19	21.00	3.46	0.16
Total Bilirubin—Day 5 (mg/dL)	2.59	1.59	1.50	0.95	0.06
Total Calcium—Day 7 (mg/dL)	8.41	0.52	7.92	0.58	0.17
INR—Day 7	1.32	0.21	1.39	0.17	0.32
Potassium—Day 7 (mmol/L)	3.91	0.40	3.83	0.31	0.56
Bicarbonate—Day 7 (mEq/L)	24.62	4.77	23.63	0.60	0.70
AST—Day 7 (U/L)	74.23	70.97	105.25	13.45	0.04
ALT—Day 7 (U/L)	194.21	228.82	179.00	60.03	0.21
BMI (kg/m^2^)	25.38	3.90	25.38	4.86	0.99
Estimated Blood Loss (mL)	6881.82	4779.78	5200.00	1307.12	0.30
Operative Time (min)	494.91	75.26	487.50	79.78	0.79
Warm Ischemia Time (min)	56.63	12.13	57.75	1.75	0.79
Cold Ischemia Time (min)	231.33	70.65	310.25	56.87	0.004
Total Ischemia Time (min)	287.96	73.14	368.00	57.75	0.004

**Table 5:** Means for Survival Time.

PRI	Mean			
	Estimate	Std. Error	95% Confidence Interval	
			Lower Bound	Upper Bound
0	89.502	5.561	78.603	100.400
1	66.796	8.956	49.243	84.350
Overall	85.764	5.050	75.866	95.662

**Table 6:** HOPE vs. DHOPE.

Parameter	HOPE	DHOPE
Perfusion route	Portal vein only	Portal vein + Hepatic artery
Temperature	4–12 °C	4–12 °C
Oxygenation	Yes	Yes
Technical complexity	Lower	Higher
Main benefit	Reduces IRI, improves mitochondrial function	Reduces IRI, improves mitochondrial function Better biliary protection
Typical indication	ECD, DCD livers	ECD, DCD

## Data Availability

Due to GDPR and institutional laws we cannot provide the datasets.

## Abbreviations

The following abbreviations are used in this manuscript:

HMP	Hypothermic Machine Perfusion
SCS	Static Cold Storage
ECD	Extended Criteria Donor
DCD	Donation after Circulatory Death
DBD	Donation after Brain Death
EAD	Early Allograft Dysfunction
UW	University of Wisconsin (solution)
HOPE	Hypothermic Oxygenated Perfusion
DHOPE	Dual Hypothermic Oxygenated Perfusion
PRI	Post-Reperfusion Injury Index
CIT	Cold ischemia time
INR	International Normalized Ratio
AST	Aspartate Aminotransferase
ALT	Alanine Aminotransferase
SD	Standard Deviation
MELD	Model for End-Stage Liver Disease
∆MAP%	Normalized difference of Mean Arterial Pressure
NAS	Non-Anastomotic Strictures
RCT	Randomized controlled trials
LDH	Lactate dehydrogenase
GGT	Gamma-glutamyl transferase
RR	Risk ratio
OR	Odd ratio
CI	Confidence interval
HCC	Hepatocellular carcinoma
HVB	hepatic virus B
HVB-D	hepatic virus B-D
HVC	hepatic virus C
ICU	intensive care unit
NASH	non alcoholic steatohepatitis
